# Determinants of Dropping Out of Remote Patient-Reported Outcome–Based Follow-Up Among Patients With Epilepsy: Prospective Cohort Study

**DOI:** 10.2196/58258

**Published:** 2025-01-14

**Authors:** Sofie Bech Vestergaard, Mette Roost, David Høyrup Christiansen, Liv Marit Valen Schougaard

**Affiliations:** 1 Department of Rheumatology, Aarhus University Hospital, Aarhus N, Denmark; 2 Department of Clinical Medicine, Aarhus University, Aarhus N, Denmark; 3 AmbuFlex, Centre for Patient-Reported Outcomes, Gødstrup Hospital, Herning, Denmark; 4 Centre for Research in Health and Nursing, Research, Regional Hospital Central Jutland, Viborg, Denmark; 5 University Clinic, Centre of Elective Surgery, Regional Hospital Silkeborg, Silkeborg, Denmark

**Keywords:** patient-reported outcome measures, dropouts, digital solutions, outpatient care, epilepsy, seizure disorder, neurological condition, cohort study, health care, Denmark, self-reported, self-management, mental health, patient satisfaction, logistic regression, social support

## Abstract

**Background:**

The use of patient-reported outcome (PRO) measures is an emerging field in health care. In the Central Denmark Region, epilepsy outpatients can participate in remote PRO-based follow-up by completing a questionnaire at home instead of attending a traditional outpatient appointment. This approach aims to encourage patient engagement and is used in approximately half of all epilepsy outpatient consultations. However, dropout in this type of follow-up is a challenging issue.

**Objective:**

This study aimed to examine the association between potential self-reported determinants and dropout in remote PRO-based follow-up for patients with epilepsy.

**Methods:**

This prospective cohort study (n=2282) explored the association between dropout in remote PRO-based follow-up for patients with epilepsy and 9 potential determinants covering 3 domains: health-related self-management, general and mental health status, and patient satisfaction. The associations were examined using multiple logistic regression analyses with adjustment for sex, age, education, and cohabitation.

**Results:**

A total of 770 patients (33.7%) dropped out of remote PRO-based follow-up over 5 years. Statistically significant associations were identified between all potential determinants and dropouts in PRO-based follow-up. Patients with low social support had an odds ratio of 2.20 (95% CI 1.38-3.50) for dropout. Patients with poor health ratings had an odds ratio of 2.17 (95% CI 1.65-2.85) for dropout. Similar estimates were identified for the remaining determinants in question.

**Conclusions:**

Patients with reduced self-management, poor health status, and low patient satisfaction had higher odds of dropout in remote PRO-based follow-up. However, further research is needed to determine the reasons for dropout.

## Introduction

Chronic conditions are a global burden that is increasing in most countries. In 2019, more than 65% of the population in Denmark had one or more chronic conditions [1]. To manage the worldwide development of these chronic conditions, it is crucial that health care resources are being optimally used. The application of telehealth initiatives could be a cost-effective solution for providing high-quality health care [2]. Over the past 50 years, the availability and use of telehealth have expanded and include various tools to manage health care [3]. The World Health Organization (WHO) defines telehealth as *“the delivery of health care services, where distance is a critical factor, by all health care professionals using information and communication technologies for the exchange of valid information for diagnosis, treatment, and prevention of disease and injuries, research and evaluation, and for the continuing education of health care providers, all in the interests of advancing the health of individuals and their communities”* [4]. After the WHO declared COVID-19 as a pandemic in March 2020, the use of telemedicine has rapidly increased [5,6]. The pandemic forced clinicians to rethink the course of treatment for their patients in various clinical settings to be effectively managed from a distance [6]. One way of using telehealth could be using patient-reported outcomes (PROs) in remote symptom monitoring. This could transform the delivery of the traditional treatment for patients with chronic conditions with prescheduled appointments by optimizing the use of resources and ensuring patient-centered care [7-10].

The use of PROs was originally developed for utilization in research but has, over time, evolved to be utilized at a patient level in clinical practice [11,12]. The use of PROs in clinical practice has been documented to improve patient survival, provide individualized care, and ensure efficient usage of health care resources [13]. In Denmark, a generic clinical PRO system for data collection in clinical practice, called AmbuFlex, offers tailor-made PRO solutions [8]. The overall purpose is to improve patient involvement and quality of treatment while optimizing resource allocation [8,9]. As of September 2023, AmbuFlex has developed 65 PRO solutions for various chronic and malignant conditions in remote outpatient care [8]. The use of PROs in remote outpatient follow-up provides real-time symptom monitoring, early detection of health issues, and rapid clinical intervention [11]. This type of follow-up requires the patient to be willing and capable of taking more responsibility as well as playing an active role in monitoring and identifying symptoms of disease [14]. Patient experiences with this course of treatment are diverse. For patients with epilepsy, it has been documented to promote health-related self-management [15]. Research on patients with rheumatoid arthritis has shown that some patients find remote PRO-based follow-up to be a flexible, time- and resource-saving solution. Others worry about the absence of face-to-face communication with a clinician and find the responsibility to cause insecurity [14].

Dropout and nonuse of telehealth solutions is a common reported challenge [16,17]. A scoping review highlights that the reasons for the nonuse of digital PRO solutions are diverse and, among others, cover the ability to use PRO, engagement, emotional issues, lack of time, and technical barriers [18]. Several contributing factors such as sex [18-21], age [19,22,23], education [18,22], and cohabitation [24-27] have been found to influence the dropout rate in digital solutions. Previous research on PRO-based follow-up for patients with epilepsy has shown that socioeconomically advantaged patients with a high level of health literacy, self-efficacy, patient activation, good well-being, and general health are more likely to be referred to PRO-based follow-up than socioeconomically disadvantaged patients [24]. Whether the same determinants apply to dropout has not been investigated. With the limited amount of literature covering dropout in remote PRO-based follow-up, further research is needed. Thus, this study aimed to investigate potential determinants for dropout in remote PRO-based follow-up among patients with epilepsy. We examined the association between dropout in PRO-based follow-up and determinants from the following three domains: health-related self-management, general and mental health status, and patient satisfaction. We hypothesized that reduced health-related self-management, poor general and mental health status, and low patient satisfaction were associated with an increased risk of dropout in PRO-based follow-up.

## Methods

### Remote PRO-Based Follow-Up for Patients With Epilepsy

PRO-based follow-up was implemented for patients with epilepsy at 3 neurological departments in the Central Denmark Region in 2012. In PRO-based follow-up, patients complete a questionnaire at home instead of having prescheduled appointments at the outpatient clinic. To attend PRO-based follow-up, patients are individually referred by a health professional. Individual referral requires that the patient must be 15 years of age or older, have no cognitive impairments, and be capable of reading and writing in Danish [28]. The questionnaire is sent at a predefined time interval of 3, 6, 12, or 24 months either electronically through a secure electronic platform or as a paper version [28].

The epilepsy questionnaire includes 47 items and deals with the frequency and intensity of seizures, medical treatment, and the patient’s well-being. The AmbuFlex system, based on an automated decision algorithm, can assess whether the patient needs clinical attention [8]. To identify patients in need of clinical attention, all response options in the questionnaire are categorized as red, yellow, or green. Red indicates that the patient needs or wants personal contact with the clinic. Yellow indicates that the patient might need personal contact. If all the patients’ answers are categorized as green, the system automatically sends a new questionnaire within the predefined period. All answers that indicate a need for clinical attention are reviewed by a nurse within 14 days. The patients’ questionnaire responses were displayed in a graphical overview available in the patients’ electronic health records. Where clinical attention is needed, the questionnaire data are used to support patient-clinician communication and clinical decision-making [28]. If a patient wants to discontinue or a clinician decides to end a PRO-based follow-up treatment, the clinician must actively register the patient as “deregistered” in the AmbuFlex system. Deceased patients are automatically deregistered in the system. PRO-based follow-ups are offered to approximately 50% of the entire patient population at the 3 neurological departments and have been used since 2012 [24]. In January 2016, approximately 3000 epilepsy outpatients attended the PRO-based follow-up program at the 3 departments.

### Study Population and Setting

We conducted a prospective cohort study among patients with epilepsy who attended PRO-based follow-ups at 3 neurology departments in the Central Denmark Region. During the period of January 1st to December 31st, 2016, patients were invited to answer a research questionnaire in addition to their usual scheduled epilepsy questionnaire from the outpatient clinic. The research questionnaire contained information about aspects related to self-management, general and mental health status, and patient satisfaction. Patients could respond to either a web-based or paper version of the questionnaire. Nonresponders received up to 3 reminders [29]. Clinicians assessed the usual epilepsy questionnaire according to their normal routine, but they were blinded to the research questionnaire.

### Outcome

The outcome was the dropout status in remote PRO-based follow-up in February 2021. This information was retrieved from the AmbuFlex system. As a clinician actively has to register the patient as “deregistered” in the AmbuFlex system, we defined dropouts in this study as “patients who, after using PRO-based follow-up, were deregistered by a clinician.” The reason for the dropout was not registered. If the patients were deregistered due to death, this information was shown in the AmbuFlex system. Patients who died during follow-up were excluded from the study.

### Potential Determinants

#### Self-Management

We assessed patient’s health-related self-management using 3 different constructs: health literacy, self-efficacy, and patient activation. Health literacy was measured using part of the multi-dimensional Health Literacy Questionnaire (HLQ) [30]. The questionnaire includes 9 subscales covering different areas of health literacy to assess strengths and challenges. The HLQ has well-documented psychometric properties considering the original questionnaire and the Danish translation, with strong content and construct validity as well as high reliability [30,31]. In this study, the following 3 subscales were used: subscale #4, “Social support for health”; subscale #6, “Ability to actively engage with health care providers,” and subscale #9, “Understanding health information well enough to know what to do.” Subscale #4 includes 5 items with a 4-point ordinal response option ranging from, 1, “strongly disagree,” 2, “disagree,” 3, “agree,” to 4, “strongly agree.” Subscales #6 and #9 both include 5 items as well a 5-point ordinal response option ranging from, 1, “cannot do,” 2, “very difficult,” 3, “quite difficult,” 4, “quite easy,” to 5, “very easy.” For all 3 subscales, a lower score indicates lower health literacy. An average score was calculated for each of the 3 subscales based on a guide provided when acquiring the license. If 1 item was missing, the mean score of the existing items was used to estimate the score. If 2 or more items were missing, the average score was not calculated. As the variables did not exhibit a log-linear relationship, the score was dichotomized into high and low health literacy. For subscale #4, high health literacy was a score >2, and low health literacy was a score ≤2. For subscales #6 and #9, high health literacy was a score >3, and low health literacy was a score ≤3.

Self-efficacy was measured using the General Self-efficacy Scale (GSES) [32]. The psychometric properties have been assessed across many nations and populations, showing a strong construct validity and a high reliability [32,33]. The GSES includes 10 questions regarding one’s belief in the ability to cope with stress and challenges. Each question has a 4-point ordinal response option ranging from 1, “not at all true,” 2, “hardly true,” 3, “moderately true,” to 4, “exactly true.” The total GSES score ranges from 10 to 40. A lower score indicates a lower degree of self-efficacy [32]. If 1 or more items were missing, the total score was not calculated. The score was included as a continuous variable.

Patient activation was assessed using part of the Patient Activation Measure (PAM) [34]. The original questionnaire includes 13 items that assessed activation in illness or health conditions [35]. In this study, 2 single items were used in a modified form: “I am confident that I can tell when I need to get outpatient care” and “I am confident I can figure out solutions when new situations or problems arise with my health condition.” Both items had 4 response options “strongly disagree,” “disagree,” “agree,” and “strongly agree.” Both items were dichotomized into strongly disagree/disagree and agree/strongly agree.

##### General and Mental Health Status

Mental well-being was measured using the WHO-5 Well-being Index (WHO-5) [36]. The WHO-5 includes 5 positively worded items focusing on mental well-being over the previous 2 weeks [36]. The questionnaire has well-documented psychometric properties for various diseases, including epilepsy [28,36,37]. Each item has a 6-point ordinal response option ranging from 5, “All the time,” 4, “most of the time,” 3, “more than half of the time,” 2, “less than half of the time,” 1, “some of the time,” to 0, “at no time.” To calculate the total score, the point for each item was added and afterward multiplied by 4, resulting in a total score between 0 and 100. A score of 100 indicates the best imaginable well-being [36]. If 1 or more items were missing, the total score was not calculated. The score was included as a continuous variable.

The patients’ general health status was assessed using a single item from Short Form-36 (SF-36) [38]. The SF-36 covers 8 different domains related to health-related quality of life [39]. It has been documented that a single item from the questionnaire can predict general health [40]. In this study, the single item “In general, would you say your health is: excellent, very good, good, fair, or poor?” was therefore used. The 5 response options were categorized into “Excellent/very good,” “good,” and “fair/poor.”

### Patient Satisfaction

The domain of patient satisfaction included patient involvement, confidence, comfort, and satisfaction. Information regarding these determinants was collected using questions inspired by the Danish Cancer Society’s Barometer Survey [41] and modified to fit the epilepsy outpatient setting. Information about patient involvement, confidence, and safety was obtained via the following questions: “Do you feel sufficiently involved in your course of treatment?,” “Are you confident that the epilepsy outpatient clinic contributes in the best possible way in your course of treatment?,” and “Are you comfortable with the follow-up you are receiving?.” These questions had five response options: “Yes, to a great extent,” “Yes, to some extent,” “To a minor extent,” “No, not at all,” or “Do not know.” Information about satisfaction was obtained through: “Overall, how would you assess your treatment in the epilepsy outpatient clinic?” with the following 5 response options: “Very good,” “Mostly good,” “Mostly bad,” “Very bad,” or “Do not know,” For all questions, the category “Do not know” was excluded in the analyses. Patient involvement, confidence, and safety were dichotomized into “Yes, to a great extent/Yes, to some extent” and “To a minor extent/not at all.” Satisfaction was dichotomized into “Very good/Mostly good” and “Mostly bad/Very bad.”

### Statistical Analyses

A Wilcoxon rank-sum test was utilized to compare age and sex between responders and nonresponders. Characteristics for the study population were presented by outcome. Continuous data were all nonnormally distributed and presented as the median and IQR. Categorical and dichotomized data were presented as numbers (n) and percentage distribution (%). The association between the potential determinants and dropouts in PRO-based follow-up was analyzed through multiple logistic regression analysis. All the odds ratio (OR) estimates were tested at a 5% significance level and reported with 95% CIs. Variables were continuously included if the linearity requirements were met. The assumption on linearity was deemed fulfilled for self-efficacy and well-being. Sex, age, education, and cohabitation were included in the adjusted analyses. The confounder variables were defined a priori based on a systematic literature review [18-27]. For the patient satisfaction domain, most of the determinants were controlled only for sex and cohabitation, as some categories had a limited number of observations. Each potential determinant was examined separately as the determinants were assessed as interdependent. All analyses were performed using the available observations giving varying observations for crude and adjusted estimates. An examination of all missing data was conducted prior to this. Upon inspection, no systematic or significant missing data were identified. For the continuous variables, postestimation analyses were performed to nuance the significance of different scores and dropouts in PRO-based follow-up. The statistical analyses were performed using Stata/IC 16.1 (StataCorp LLC).

### Ethical Considerations

The study was approved by the Danish Data Protection Agency (number 1-16-02-691-14). The Ethics Committee of the Central Denmark Region was consulted and decided that the study did not require their approval. According to Danish law, only studies involving human biological material require committee approval. Correspondence with the ethics committee is available from the authors upon request. Informed consent for participation in the study was obtained in accordance with guidelines from the Danish Data Protection Agency. Information was provided to patients in a letter along with the questionnaire. Patients were informed that responding to the questionnaire constituted active consent for their participation and the use of their data in the research project. They were also informed that they could withdraw their consent at any time. All data were stored and handled with confidentiality.

## Results

### Patient Characteristics

**Figure 1.**
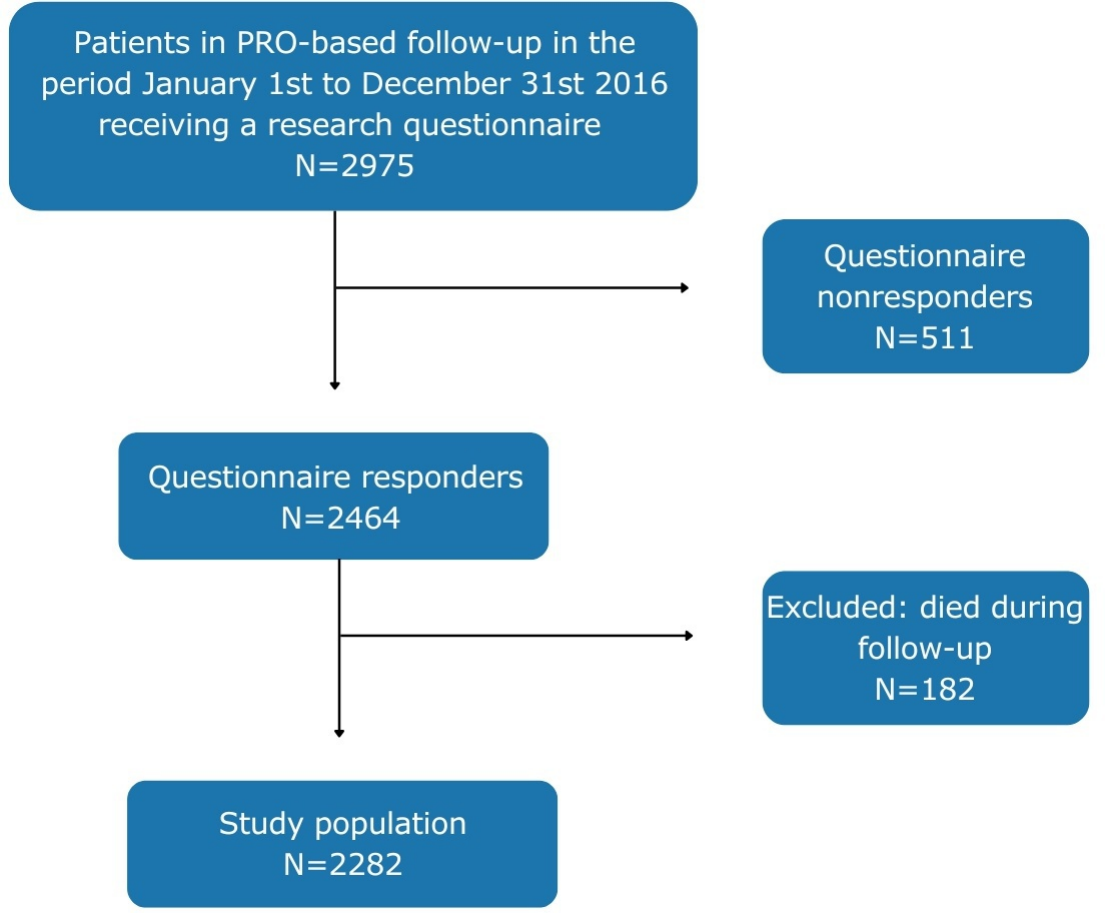
Flowchart of patients included in the study.

From January 1st to December 31st, 2016, a total of 2975 patients received a paper or web-based questionnaire. Of these, 2464 patients answered the questionnaire, resulting in a response rate of 83%. During the follow-up period, 182 patients died. The final study population included 2282 patients (Figure 1). The median age in the study population was 49 (IQR 29.7) years, 1125 (49.3%) were male, 982 (43%) had a low education, and 1645 (72.1%) were not living alone (Table 1). A total of 770 patients (33.7%) had dropped out of PRO-based follow-up in February 2021. No statistical differences were found between responders and nonresponders in sex distribution (*P*=.34) or age distribution (*P*=.70). Missing data ranged from 1.5% (general health) to 7.8% (general self-efficacy) (Table 1).

**Table 1.** Baseline characteristics and patient-reported potential determinants by status in patient-reported outcome (PRO)–based follow-up among patients with epilepsy in February 2021.

Characteristics	Total, n=2282	Active, n=1512	Dropout, n=770
Sex, n (%)			
Male	1125 (49.3)	757 (50.1)	368 (47.8)
Female	1157 (50.7)	755 (49.9)	402 (52.2)
Age, years, n (%)			
15‐24	245 (10.7)	128 (8.5)	117 (15.2)
25‐39	538 (23.6)	340 (22.5)	198 (25.7)
40‐54	586 (25.7)	407 (26.9)	179 (23.3)
55‐69	579 (25.4)	421 (27.8)	158 (20.5)
≥70	334 (14.6)	216 (14.3)	118 (15.3)
Education, n (%)			
None	512 (22.4)	317 (21.0)	195 (25.3)
Low	982 (43.0)	674 (44.6)	308 (40.0)
Medium/high	622 (27.3)	426 (28.2)	196 (25.5)
Missing	166 (7.3)	95 (6.3)	71 (9.2)
Cohabitation, n (%)			
Living alone	526 (23.1)	327 (21.6)	199 (25.8)
Not living alone	1645 (72.1)	1126 (74.5)	519 (67.4)
Missing	111 (4.9)	59 (3.9)	52 (6.8)
Health literacy #4^a^, n (%)			
Low ≤2	77 (3.4)	37 (2.5)	40 (5.2)
High >2	2049 (89.8)	1395 (92.3)	654 (84.9)
Missing	156 (6.8)	80 (5.3)	76 (9.9)
Health literacy #6^b^, n (%)			
Low ≤3	398 (17.4)	222 (14.7)	176 (22.9)
High >3	1728 (75.7)	1209 (80.0)	519 (67.4)
Missing	156 (6.8)	81 (5.4)	75 (9.7)
Health literacy #9^c^, n (%)			
Low ≤3	323 (14.2)	172 (11.4)	151 (19.6)
High >3	1796 (78.7)	1253 (82.9)	543 (70.5)
Missing	163 (7.1)	87 (5.8)	76 (9.9)
Self-efficacy			
General Self-efficacy Scale score, median (IQR)	30 (25‐33)	30 (26‐34)	29 (24‐32)
Missing, n (%)	177 (7.8)	91 (6.0)	86 (11.2)
PAM #5^d^, n (%)			
Strongly disagree/disagree	221 (9.7)	118 (7.8)	103 (13.4)
Agree/strongly agree	1926 (84.4)	1323 (87.5)	603 (78.3)
Missing	135 (5.9)	71 (4.7)	64 (8.3)
PAM #12^e^, n (%)			
Strongly disagree/disagree	342 (15.0)	193 (12.8)	149 (19.4)
Agree/strongly agree	1797 (78.8)	1246 (82.4)	551 (71.6)
Missing	143 (6.3)	73 (4.8)	70 (9.1)
Mental well-being			
WHO-5 Well-being Index score, median (IQR)	76 (60‐80)	76 (64‐80)	72 (52‐80)
Missing, n (%)	64 (2.8)	35 (2.3)	29 (3.8)
General health^f^, n (%)			
Excellent/very good	1030 (45.1)	719 (47.6)	311 (40.4)
Good	886 (38.8)	597 (39.5)	289 (37.5)
Fair/poor	333 (14.6)	181 (12.0)	152 (19.7)
Missing	33 (1.5)	15 (1.0)	18 (2.3)
Patient involvement, n (%)			
A great extent/some extent	1820 (79.8)	1259 (83.3)	561 (72.9)
A lesser extent/not at all	189 (8.3)	102 (6.8)	87 (11.3)
Do not know	140 (6.1)	82 (5.2)	58 (7.5)
Missing	133 (5.8)	69 (4.6)	64 (8.3)
Confidence, n (%)			
A great extent/some extent	1945 (85.2)	1324 (87.6)	621 (80.7)
A minor extent/not at all	99 (4.3)	56 (3.7)	43 (5.6)
Do not know	116 (5.1)	68 (4.5)	48 (6.2)
Missing	122 (5.4)	64 (4.2)	58 (7.5)
Comfort, n (%)			
A great extent/some extent	1896 (83.1)	1307 (86.4)	589 (76.5)
A minor extent/not at all	108 (4.7)	56 (3.7)	52 (6.8)
Do not know	148 (6.5)	80 (5.3)	68 (8.8)
Missing	130 (5.7)	69 (4.6)	61 (7.9)
Satisfaction, n (%)			
Very good/mostly good	1863 (81.6)	1288 (85.2)	575 (74.7)
Mostly bad/very bad	93 (4.1)	49 (3.2)	44 (5.7)
Do not know	197 (8.6)	106 (7.0)	91 (11.8)
Missing	129 (5.7)	69 (4.6)	60 (7.8)

a Health Literacy Questionnaire subscale #4 “Social support for health.”

b Health Literacy Questionnaire subscale #6 “Ability to actively engage with health care providers.”

c Health Literacy Questionnaire subscale #9 “Understanding health information well enough to know what to do.”

d Patient Activation Measure item #5: “I am confident that I can tell when I need to get outpatient care.”

e Patient Activation Measure item #12: “I am confident I can figure out solutions when new situations or problems arise with my health condition.”

f Assessed using a single item from Short Form-36.

### Associations Between Self-Management and Dropout in PRO-Based Follow-Up

The ORs for the self-management determinants and dropout in PRO-based follow-up are presented in Table 2. Statistically significant associations were found between all potential self-management determinants and dropout. A lower degree of self-management was associated with dropout. Patients reporting having low social support for health (HLQ4) had higher odds of dropout (OR 2,02, 95% CI 1.38-3.50). Similar associations were identified for the remaining determinants regarding health literacy (Table 2). Patients who had lower confidence in knowing when to get outpatient care (PAM5) had higher odds of dropout (OR 1.94, 95% CI 1.44-2.62). A high level of self-efficacy had decreased odds for dropout per point (OR 0.96, 95% CI 0.95-0.98). When comparing a patient with the lowest GSES score (score of 10) to a patient with the highest possible GSES score (score of 40), the patient with the low self-efficacy had an OR of 3.21 (95% CI 2.05-5.03) for dropout (Table 3).

**Table 2.** Crude and adjusted odds ratio (OR) for potential determinants of self-management and drop-out in patient-reported outcome (PRO)–based follow-up among patients with epilepsy.

Potential determinants	Crude		Adjusted^a^	
	OR (95% CI)	*P* value	OR (95% CI)	*P* value
Social support for health (HLQ 4)	n=2126		n=2099	
High >2	ref		ref	
Low ≤2	2.31(1.46-3.64)	<.001	2.20 (1.38-3.50)^b^	<.001
Ability to actively engage with health care providers (HLQ 6)	n=2126		n=2050	
High >3	ref		ref	
Low ≤3	1.85 (1.48-2.31)	<.001	1.82 (1.44-2.31)	<.001
Low ability to understand health information well enough to know what to do (HLQ9)	n=2119		n=2046	
High >3	ref		ref	
Low ≤3	2.03 (1.59-2.58)	<.001	2.15 (1.65-2.80)	<.001
Self-efficacy	n=2105		n=2032	
General Self-efficacy Scale score ^c^	0.96 (0.95-0.98)^d^	<.001	0.96 (0.95-0.98)^d^	<.001
PAM #5	n=2147		n=2066	
Strongly agree/agree	ref		ref	
Disagree/strongly disagree	1.92 (1.45-2.54)	<.001	1.94 (1.44-2.62)	<.001
PAM #12	n=2139		n=2060	
Strongly agree/agree	ref		ref	
Disagree/strongly disagree	1.75 (1.38-2.21)	<.001	1.84 (1.43-2.36)	<.001

a Adjusted for sex, age, education, and cohabitation.

b Adjusted for sex and cohabitation due to low observations in some categories.

c General Self-Efficacy Scale score in the interval 10‐40.

d Per point increase in the General Self-Efficacy Scale score. The score 0 is reference.

**Table 3.** Adjusted odds ratio (OR) for dropout in patient-reported outcome (PRO)–based follow-up among patients with epilepsy at different scores relative to a patient with the highest possible score (40) on the General Self-Efficacy Scale.

General Self-efficacy scale score	OR (95% CI)^a^	*P* value
40 points	ref	
30 points	1.47 (1.27-1.71)	<.001
20 points	2.18 (1.61-2.94)	<.001
10 points	3.21 (2.05-5.03)	<.001

a OR for dropout relative to a reference with a score on 40 adjusted for sex, age, education, and cohabitation

### Associations Between General and Mental Health Status and Dropout in PRO-Based Follow-Up

We found that patients with good general health status had statistically significantly higher odds for dropout (OR 1.28, 95% CI 1.65-2.85) compared to patients who reported excellent/very good health status. Similarly, patients with fair/poor general heath status had statistically significantly higher odds for dropout (OR 2.17, 95% CI 1.65-2.85) compared to patients who reported excellent/very good health status (Table 4). Moreover, patients with lower mental health status had higher odds of dropout. A patient with the lowest possible WHO-5 score (0) had an OR of 3.16 (95% CI 1.94-5.15) for dropout relative to a patient with the highest score (100) (Table 5).

**Table 4.** Crude and adjusted Odds ratio (OR) for potential determinants of general and mental health status and drop-out in PRO-based follow-up among patients with epilepsy.

Potential determinants	Crude		Adjusted^a^	
	OR (95% CI)	*P* value	OR (95% CI)	*P* value
Mental well-being	n=2218		n=2063	
WHO-5 Well-being Index score^b^	0.99 (0.98-0.99)^c^	<.001	0.99 (0.98-0.99)^c^	<.001
General health (SF-36)^d^	n=2249		n=2088	
Excellent/very good	ref		ref	
Good	1.12 (0.92-1.36)	.25	1.28 (1.04-1.57)	.02
Fair/poor	1.94 (1.51-2.50)	<.001	2.17 (1.65-2.85)	<.001

a Adjusted for sex, age, education, and cohabitation.

b WHO-5 Well-being Index score in the interval 0‐100.

c Per point increase in the WHO-5 Well-being Index score. The score 0 is reference.

d Using a single item from the Short Form-36.

**Table 5.** Adjusted odds ratio (OR) for dropout in patient-reported outcome (PRO)-based follow-up among patients with epilepsy at different scores relative to a patient with the highest possible score (100) on the WHO-5 Well-being Index score.

WHO-5 Well-being Index score	OR (95% CI)^a^	*P* value
0 points	3.16 (1.94-5.15)	<.001
25 points	2.37 (1.64-3.42)	<.001
50 points	1.78 (1.39-2.27)	<.001
75 points	1.33 (1.18-1.51)	<.001

a OR for dropout relative to a reference with a score on 100 adjusted for sex, age, education, and cohabitation.

### Associations Between Patient Satisfaction and Dropout in PRO-Based Follow-Up

The associations between patient satisfaction and dropout in PRO-based follow-up are presented in Table 6. Patients who, to a minor extent/not at all, felt sufficiently involved in the course of treatment had an OR of 1.93 (95% CI 1.42-2.63) of dropping out compared to patients who felt sufficiently involved. Similar associations were found for the determinants of confidence and comfort. Patients who were not satisfied and found their overall treatment to be mostly bad/very bad had an OR of 2.00 (95% CI: 1.31;3.06) for dropout compared to satisfied patients.

**Table 6.** Crude and adjusted odds ratio (OR) for potential determinants for patient satisfaction and drop-out in patient-reported outcome (PRO)–based follow-up among patients with epilepsy.

Potential determinants	Crude		Adjusted^a^	
	OR (95% CI)	*P* value	OR (95% CI)	*P* value
Patient involvement	n=2009		n=1935	
A great extent/some extent	ref		ref	
A minor extent/not at all	1.91 (1.41-2.59)	<.001	1.93(1.42-2.63)^b^	<.001
Confidence	n=2044		n=2021	
A great extent/some extent	ref		ref	
A minor extent/not at all	1.64 (1.09-2.46)	.02	1.62 (1.07-2.44)	.02
Comfort	n=2004		n=1981	
A great extent/some extent	ref		ref	
A minor extent/not at all	2.06 (1.40-3.04)	<.001	2.04 (1.38-3.02)	<.001
Satisfaction	n=1956		n=1934	
Very good/mostly good	ref		ref	
Mostly bad/very bad	2.01(1.32-3.06)	<.001	2.00 (1.31-3.06)	<.001

a Adjusted for sex and cohabitation.

b Adjusted for sex, education, and cohabitation.

## Discussion

### Principal Findings and Comparison With Previous Works

The majority of the patients reported a high level of self-management, good health status, and a high degree of patient satisfaction. A strong association was found between all 9 self-reported potential determinants and dropout in remote PRO-based follow-up for patients with epilepsy in the Central Denmark Region. Patients with reduced self-management, poor health status, and low patient satisfaction had higher odds of dropout in PRO-based follow-up. The association appeared independent, as the correction for sex, age, education, and cohabitation showed only a minor impact on the relation between the potential determinants and dropout.

To the best of our knowledge, previous research has not primarily focused on investigating dropout rates; instead, dropout has typically been included as a secondary finding [18,22]. Furthermore, past research has not given exclusive attention to PRO-based follow-up but has focused on telehealth more broadly, encompassing app-based interventions [18,22]. Using a meta-analysis, Meyerowitz-Katz et al determined that app-based chronic disease management interventions exhibit a dropout rate of 49% (95% CI 27%-70%) [22]. A scoping review of 31 studies on patient reasons for not using digital PRO concepts identified a dropout range of 2.4% to 72.3% [18]. Both studies reported higher dropout rates compared to the dropout rate of 33.7% in this study. The studies included follow-up periods with varying response frequencies, ranging from daily to every 3 months [18,22]. This variability in follow-up schedules may have contributed to the higher dropout rates observed in the comparative studies, along with the contextual differences between dropout rates in digital interventions and dropout rates in continuous chronic disease follow-up. Adherence to technology is another concept that can indicate how patients use digital solutions. A recently published systematic review investigated factors associated with adherence to tele-monitoring using electronic PRO measures in patients with chronic diseases, including heart failure, rheumatoid arthritis, and chronic pain [42]. This review found adherence rates to range between 61% and 96% in the included studies [42], which aligns with the dropout rate of approximately 30% found in our study.

Patient characteristics associated with dropout have been investigated mainly in relation to sociodemographic factors, such as sex, age, education, and cohabitation status in other studies [18,22,42]. Self-reported aspects, including self-management and health literacy, have to our knowledge not been reported in other studies regarding the dropout rate in digital interventions. Thus, our study contributes novel knowledge in this field and underlines the importance of considering different individual needs and competencies when implementing digital solutions in clinical care. Nielsen et al also investigated reasons for not using digital PRO concepts in clinical practice among patients with long-term conditions. They found several reasons for digital nonuse of PRO; among others, the themes covered the ability to use PRO data, engagement, emotional distress, and technical barriers [18]. From the patient’s point of view, these reasons are important to consider before implementing a digital PRO solution in a clinical setting. Reasons for dropout in PRO-based follow-up will be further investigated in future research by interviewing patients as well as clinicians.

The clinicians’ perspective is important to investigate because dropout could be related not only to digital skills or engagement by patients. From the health care provider’s point of view, there may also be concerns that arise during the treatment of patients included in a digital solution. Qualitative research regarding the clinicians’ perspective of using PRO-based follow-up in patients with epilepsy has shown reluctance among clinicians [43]. For example, some of the clinicians found the lack of interpersonal contact as a negative consequence of PRO-based follow-up and felt unsure about some of the patients’ capabilities to participate even though the patients were already included in PRO-based follow-up [43]. There is no standardized guideline regarding referral to PRO-based follow-up in patients with epilepsy in the Central Denmark Region; thus, the personal preferences amongst clinicians may play a role when deciding to refer or exclude patients from PRO-based follow-up. Prior research investigating factors associated with referral to PRO-based follow-up among patients with epilepsy and patients with rheumatoid arthritis supports this statement since patients with a higher socioeconomic background more often were referred to PRO-based follow-up compared to patients with a lower socioeconomic background [24,44]. Similar to our finding, the study in patients with epilepsy also found a positive association between a high level of health literacy and referral to PRO-based follow-up [24]. These findings in addition to our current study support the importance on focusing on the involvement of vulnerable patient groups when designing digital PRO solutions in clinical practice.

### Strengths and Limitations

We conducted a large Danish prospective cohort study among a representative epilepsy population enrolled in remote PRO-based follow-up. Self-reported data were collected before the outcome of interest; thus, potential misclassification would be nondifferential and not bias the results. The outcome (dropout) was register-based, and missing data were acceptable for both self-reported and register-based data. Thus, the internal and external validity of this study are considered sufficient. However, some limitations should be noted. First, we possessed only baseline information about the potential determinants. It is possible that the patients’ perceptions of self-management, health status, and patient satisfaction changed during the follow-up period.

Second, the initial response rate in this study was high (83%); however, the only data available regarding the nonrespondents were sex and age. No differences were found between responders and nonresponders, but we cannot rule out that nonresponders did differ from the respondents considering other characteristics. In general, we found high levels of self-management, health status, and satisfaction amongst the responders. Nondifferential selection bias could occur if a lower level of these aspects may have been reported by the nonresponders. Third, an issue occurred with the interpretation of the question regarding the confounder variable education. This was evident for some younger patients who reported having a higher educational level than plausible for their age. It was not possible to determine if this issue was present throughout the study, and there is a possibility that this misclassification may have contributed to insufficient correction for education. Furthermore, despite adjusting for the most common sociodemographic confounders, a risk of residual confounding cannot be completely ruled out. Finally, differentiating the reasons for dropout would have enhanced the strength of this study. However, this information was not available and will be explored in future research.

### Conclusions

In this study, we found that reduced self-management, poor health status, and patient dissatisfaction were associated with an increased odds of dropout in remote PRO-based follow-up. Thus, findings from this study support that a remote approach in outpatient follow-up may not be suitable to the whole patient population, but a selected group of patients. However, further research is necessary to explore the reasons for patient dropout. The findings underline the importance of involving patients with different needs and competencies when designing and implementing digital PRO solutions in the health care system.

## Abbreviations

CI: confidence interval
GSES: General Self-Efficacy Scale
HLQ: Health Literacy Questionnaire
IQR: Interquartile range
OR: odds ratio
PAM: patient activation measure
PRO: patient-reported outcome
SF-36: Short Form-36
WHO: World Health Organization
WHO-5: WHO-5 Well-being Index
